# Not All Chubby Cheeks Are Cute: A Case of Cherubism

**DOI:** 10.7759/cureus.65841

**Published:** 2024-07-31

**Authors:** Apurvaa Pachva, Krishna Teja Narella, Alekhya A Reddy, Sai Pavan Kumar

**Affiliations:** 1 Department of Radiology, Dr. D. Y. Patil Medical College, Hospital and Research Centre, Dr. D. Y. Patil Vidyapeeth (Deemed to be University), Pune, IND

**Keywords:** fibrous tissue, mandible swelling, rare genetic disorder, autosomal dominant disorder, cherubism

## Abstract

The growth of the jaw occurs painlessly in cherubism, a rare genetic disorder where normal bone is replaced by fibrous tissue and undeveloped bone. Usually running in families, this non-cancerous genetic condition naturally reaches a limit and stops growing. The main characteristic is the aberrant growth of osseous and fibrous tissue in the maxilla and mandible, which is frequently seen in children. Cherubism is inherited autosomal dominantly, though reports have included individuals without a family history. The disorder has specific radiographic and histological features that drastically affect facial appearance. This article provides a thorough case study of a male 16-year-old with cherubism, emphasizing management techniques and clinical and radiological results. Radiological imaging is essential for diagnosis and management because it can identify the distinctive features of cherubism and the treatment implications associated with it.

## Introduction

Cherubism is an autosomal dominant rare genetic disorder affecting the jawbones that gets its name from its resemblance to cherubic portrayals in art. Early infancy is when it typically shows up as painless bilateral jaw enlargement [1,2]. Despite its known inheritance as an autosomal dominant disorder, few reports have included individuals without a family history [3-5]. Mutations in the SH3 domain binding protein 2 (2SH3BP2) gene result in aberrant bone remodeling and the development of multinucleated large cells in the mandible, which cause cherubism [6]. The diagnosis and monitoring of cherubism depend heavily on CT and MRI, which also help in treatment planning by giving specific details on lesion size and bone involvement [7-9].

## Case presentation

**Figure 1:**
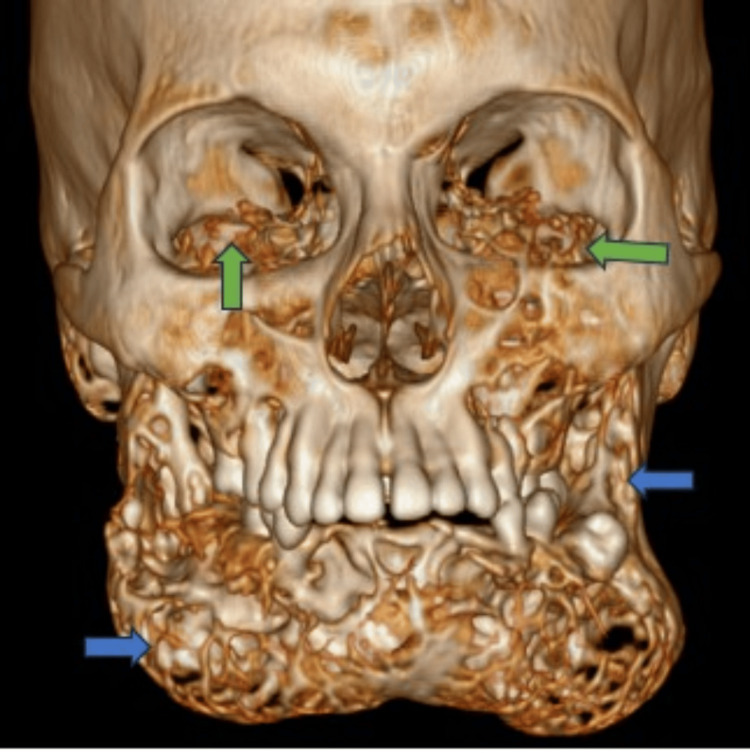
Three-dimensional CT image showing an expansile lytic lesion affecting the mandible (blue arrows) and maxilla (green arrows) CT: computed tomography

A male 16-year-old patient had a gradual, painless growth of his jaw that started in early childhood. Both sides of the growth had no pain, but they restricted the nasal canal and gave the eyes an upward-staring aspect. Significant edema at the mandibular angles was found on both sides of the lower jawbone during the clinical examination. The patient was born into a healthy, second-degree consanguineous marriage. A CT face examination showed multiple well-defined, multilocular expansile lytic lesions predominantly involving the mandible on both sides (Figure 1).

**Figure 2:**
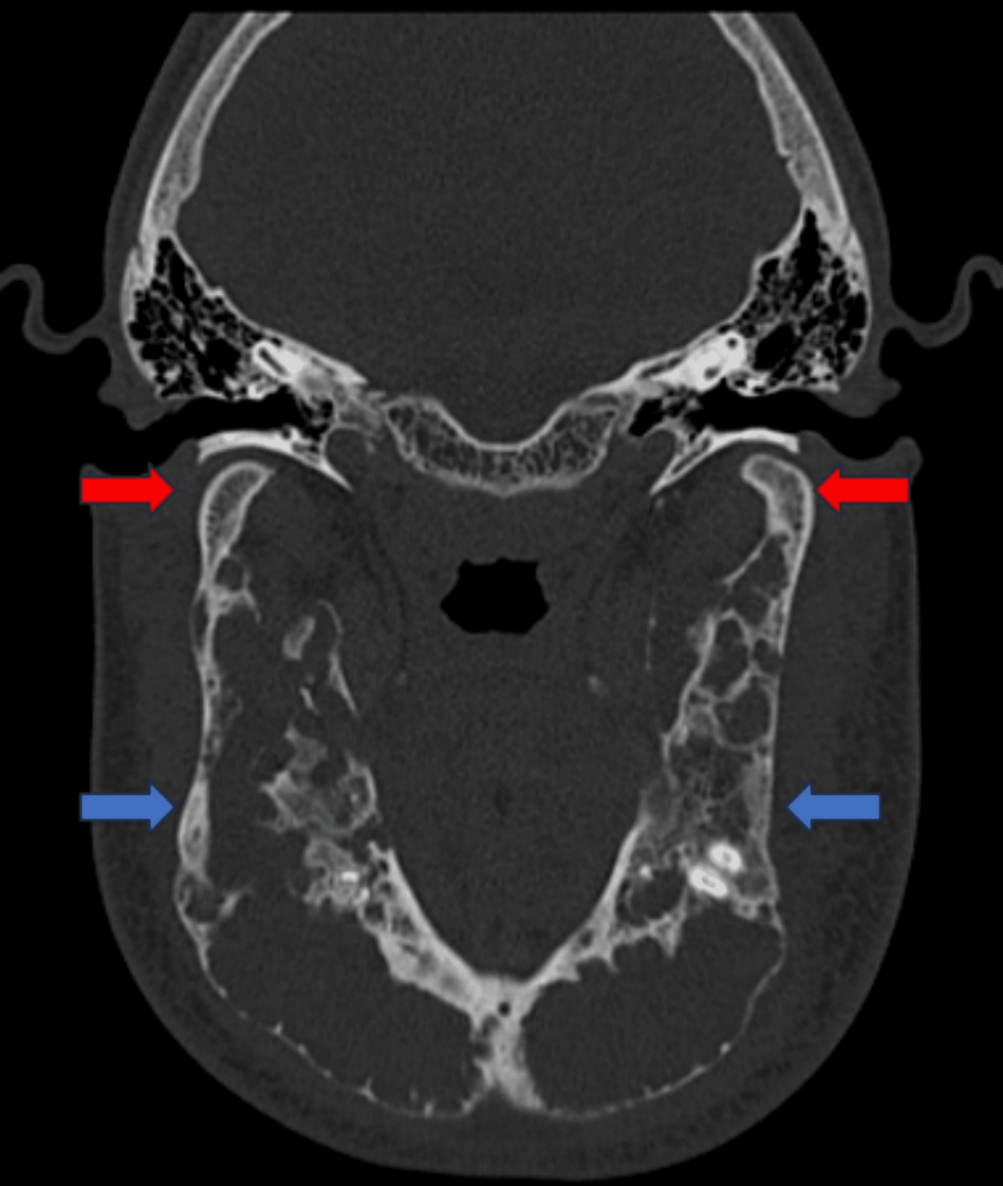
CT face bone window axial view showing multiple well-defined, multilocular expansile lytic lesions noted in the symphysis, body, rami, and coronoid processes of the mandible (blue arrows) and sparing of condyles (red arrow) CT: computed tomography

The symphysis, body, rami, and coronoid processes of the mandible were all affected by the lesion; the condyles were unaffected. There was expansile remodeling of the mandible, with thinning of the adjacent cortex and coarse internal trabeculae giving a multilocular appearance. Cortical erosions at multiple places were seen. No adjacent periosteal reaction was noted. Adjacent soft tissue thickening, or edema, was noted (Figure 2).

**Figure 3:**
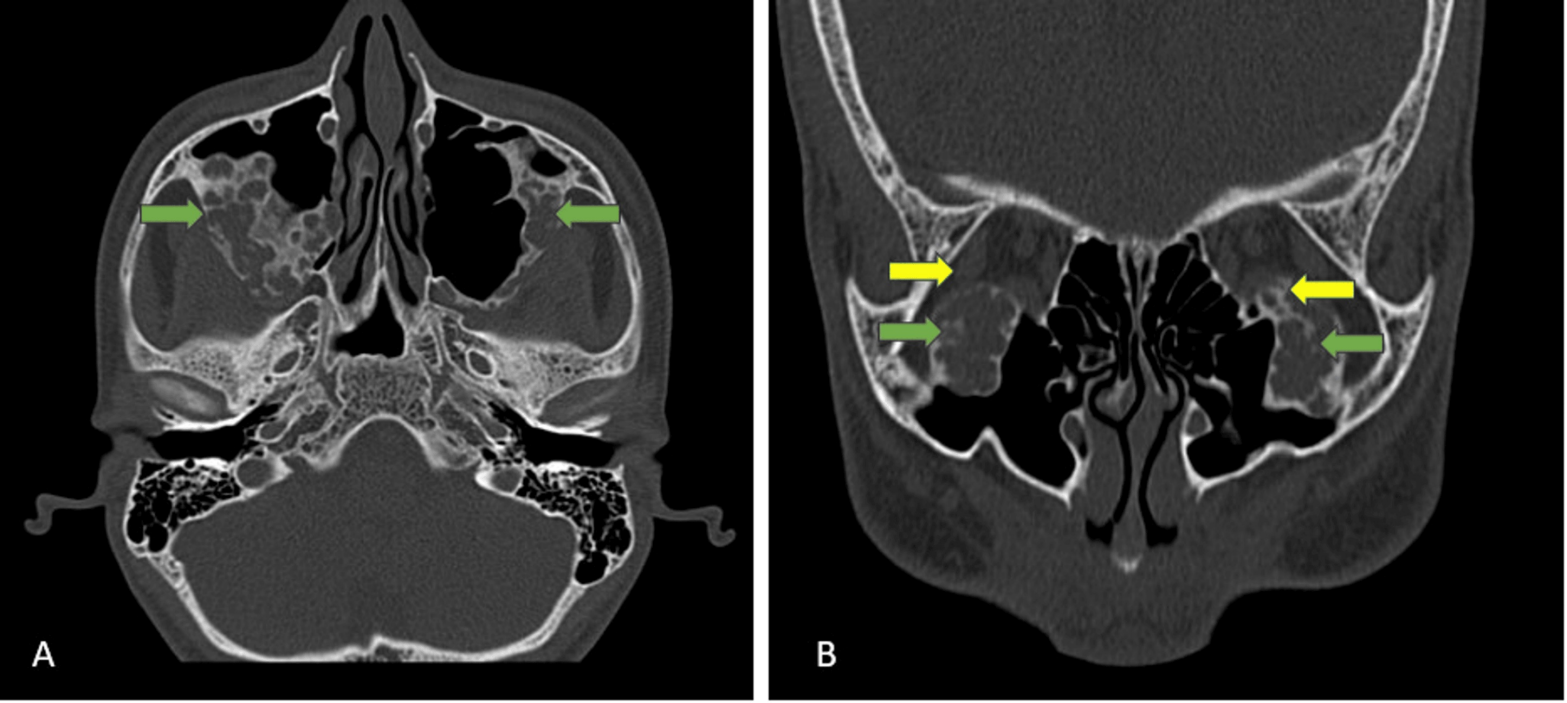
CT face bone window (A) axial view and (B) coronal view showing multilocular expansile lytic lesions involving the posterolateral wall and roof of the bilateral maxillae (green arrows). The expanded roof of the maxillae bulged and encroached upon the bilateral orbits, displacing the inferior recti bilaterally and the right lateral recti muscles (yellow arrow) CT: computed tomography

Similar, well-defined, multilocular expansile lytic lesions were also noted involving the posterolateral wall and roof of the bilateral maxillae. These lesions showed soft tissue density within (Figures 3A, 3B).

The expanded mandible and maxillae indented the adjacent soft tissue structures, encroaching upon the bilateral infratemporal fossae. The expanded roof of the maxillae bulged and encroached upon the bilateral orbits, displacing the inferior recti and, to a lesser extent, the right lateral recti muscles (Figure 3B).

**Figure 4:**
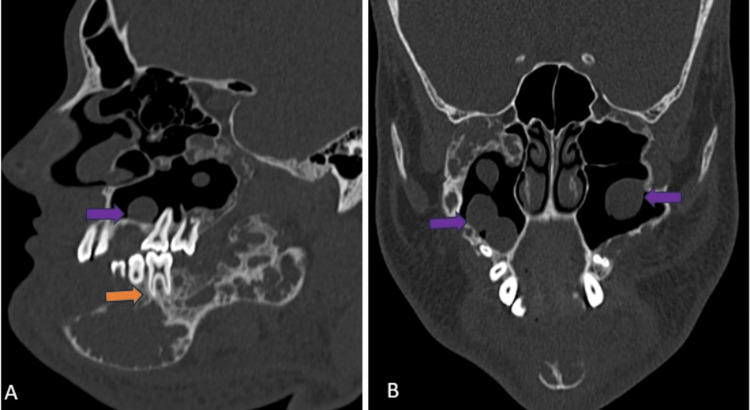
CT face bone window (A) sagittal view and (B) coronal view showing mandibular lesions extending into the alveolar arch, involving the roots of the lower teeth (orange arrow) and mucosal density and polypoidal thickening in the bilateral maxillary sinuses (violet arrow) CT: computed tomography

The mandibular lesions extended into the alveolar arch, involving the roots of the lower teeth (Figure 4A, 4B). No obvious pathological fractures were noted. Mucosal density and polypoidal thickening were observed in the bilateral maxillary sinuses (Figure 4A, 4B). A few prominent cervical lymph nodes were seen bilaterally.

## Discussion

A rare genetic condition called cherubism results in painless enlargement of the jaw because it replaces immature bone with fibrous tissue [1]. The illness appears in early childhood and is inherited in an autosomal dominant way, varying in severity. Discrete, multilocular lytic lesions primarily affect the mandible and maxilla due to mutations in the SH3BP2 gene, which is essential for osteoclast activity and bone remodeling [10]. In clinical settings, cherubism manifests as lower jaw muscle hypertrophy, which causes asymmetry in the face and difficulty chewing food. Airway obstruction may result in severe cases. Characteristic multilocular, expansile radiolucent lesions with well-defined boundaries, cortical thinning, and soft tissue density can be seen on CT and MRI images. This case showed significant invasion of soft tissue, cortical bone degradation, and involvement of the mandible and maxilla. MRI provides detailed pictures of soft tissue involvement, which helps with surgical planning [9].

From a histological perspective, cherubism is characterized by the replacement of normal bone with fibrous tissue, juvenile braided bone, and multinucleated giant cells. The fibro-osseous transition is responsible for the "soap bubble" or "ground glass" lesions seen on radiographs. The differential diagnosis consists of central giant cell granuloma, fibrous dysplasia, and ossifying fibroma. A histological investigation is necessary to diagnose cherubism and distinguish it from other disorders that are similar to it [8].

Since cherubism frequently resolves on its own during adolescence, conservative therapy includes routine clinical and radiographic surveillance to track lesion stability and progression. In order to restore facial symmetry and relieve pressure on important tissues, surgical intervention is taken into consideration when there is a considerable functional impairment or cosmetic problem [10,11]. Given that cherubism is inherited autosomal dominant with varied expression, genetic counseling is essential for families. Affected individuals have a 50% chance of passing on the illness to each of their offspring. For families with known variations in the SH3BP2 gene, preimplantation genetic testing and prenatal diagnostics are available alternatives. Subsequent investigations have to concentrate on comprehending the molecular processes of SH3BP2 mutations and their influence on bone remodeling, employing genetic sequencing technologies to facilitate prompt diagnosis and customized therapy [12].

## Conclusions

Cherubism is an uncommon hereditary condition with unique radiological and clinical characteristics. Optimizing patient outcomes requires prompt diagnosis and thorough care from radiologists, oral surgeons, geneticists, and orthodontists. For the purpose of determining the extent of the lesion, making therapeutic decisions, and diagnosing cherubism, radiological imaging techniques like CT and MRI are necessary. A thorough investigation into the genetic foundation and pathophysiology of cherubism will improve our comprehension and treatment of this intricate illness.
